# How do support networks influence physical activity participation among adults with a learning disability? A scoping review protocol

**DOI:** 10.1136/bmjopen-2026-117713

**Published:** 2026-10-23

**Authors:** Ellie Rossiter, Anna Pettican, Paul Freeman, James Black, Simone Coetzee, Ewen Speed

**Affiliations:** 1 University of Essex, Colchester, UK; 2 School of Health and Social Care, University of Essex, Colchester, Essex, UK

**Keywords:** Disabled Persons, Social Support, Caregivers, Exercise

## Abstract

**Abstract:**

**Introduction:**

Adults with a learning disability experience enduring health inequalities, including lower physical activity participation compared with the general population.

Support networks are central to the lives of many adults with a learning disability and play an influential role in everyday health behaviours. Although support networks are increasingly recognised as important, evidence and thorough understanding of the relational and structural dynamics of support networks in facilitating physical activity remain limited.

**Methods and analysis:**

This scoping review protocol outlines a systematic approach to mapping literature examining how support networks influence physical activity participation among adults with a learning disability.The review seeks to explore three research questions shaped by the population, concept and context framework to explore: (1) Who and what are the key drivers or limiters within support networks in relation to physical activity participation for adults with a learning disability? (2) How do those within and across support networks (eg, families, professional caregivers and advocacy groups) interact, collaborate and influence each other in promoting physical activity for adults with a learning disability? (3) How do adults with a learning disability engage in decision-making within their support networks in relation to physical activity and to what extent do they have power and autonomy in these relationships? A comprehensive search of social science and physical activity databases will be conducted to retrieve relevant literature. Title, abstract and full-text review will be conducted independently by two reviewers, ER and JB. Data will be extracted in tabular format, and synthesised and described in relation to the research questions. ER will engage in a consultation stage, informed by principles of participatory research, to share preliminary findings with adults with a learning disability and members of their support networks to strengthen practical relevance and interpretation.

**Ethics and dissemination:**

This review protocol does not require ethical approval as primary data have not been collected/analysed. The scoping review will deepen our understanding of using support networks to promote and facilitate physical activity with adults with a learning disability and highlight opportunities and recommendations to inform future research. Findings will be disseminated via publications, presentations as well as in accessible forums and stakeholder meetings.

**STRENGTHS AND LIMITATIONS OF THIS STUDY**

- A deliberate decision was made to search databases that span sport and physical activity, and health and social care, to ensure a diversity of literature with relevance to the topic was identified.
- We will include a participatory engagement element in this review, including sharing the protocol with people across disciplines and backgrounds, as well as engaging with people with a learning disability and members of their support network, to develop co-produced practical insights for enhanced applicability.
- We are aware that concepts such as *learning disability*, *social support* and *physical activity* may be defined and conceptualised differently across disciplines, countries, academic fields and sectors, which could lead to challenges in comparing or categorising evidence consistently.

## Objective

 The objective of this scoping review protocol is to provide a comprehensive plan to conduct a scoping review with the aim of mapping the existing literature relating to the role of support networks in promoting physical activity for adults with a learning disability. The completed review will provide a comprehensive overview of how support networks influence participation in physical activity among adults with a learning disability. To achieve this, we seek to explore three review questions.

## Review questions

1. Who and what are the key drivers or limiters within support networks in relation to physical activity participation for adults with a learning disability?
2. How do those within and across support networks (eg, families, professional caregivers and advocacy groups) interact, collaborate and influence each other in promoting physical activity for adults with a learning disability?
3. How do adults with a learning disability engage in decision-making within their support networks in relation to physical activity and to what extent do they have power and autonomy in these relationships?

## Introduction

People with a learning disability face persistent and well-documented health inequalities, experiencing significantly poorer health outcomes and reduced life expectancy compared with the general population.^1 2^ Lower levels of physical activity are a contributing factor to these health disparities, with individuals with a learning disability often participating in physical activity less frequently and less intensively than their peers without disabilities.^3 4^ Physical activity plays a critical role in improving cardiovascular health, mental well-being and quality of life.^5 6^ However, people with a learning disability may face unique challenges, including structural, social and environmental barriers, which continue to restrict access and engagement in health-related behaviours, such as physical activity.^7 8^

Support networks are often central to the lives of many adults with a learning disability. These networks vary between individuals but frequently comprise families, paid carers, support workers, friends, peers and community organisations.^9–11^ Support networks may provide practical, emotional and social support and often act as key influencers in everyday activities, including opportunities to be physically active.^12^ This influence can be both enabling and limiting: while some networks actively encourage participation and inclusion, others may unintentionally restrict opportunities due to risk aversion, lack of awareness or organisational constraints.^7^

In the context of learning disability research, the role of support networks is increasingly recognised as vital, yet this complex topic is still under-represented.^10 13^ Exploring the role of support networks in research is therefore essential to gaining insight into how decisions are made, how physical activity is facilitated or hindered and how inclusive practices can be promoted across settings.^14^ By deepening our understanding of how support networks can shape access to and participation in physical activity, we can begin to shape collaborative support strategies to facilitate health-promoting behaviours, thus reducing health inequalities.^15^

The scoping review is an element of a larger overarching participatory research project with adults with a learning disability. The purpose of this scoping review is to systematically map the existing literature on how support networks influence physical activity participation among adults with a learning disability. A scoping review has been selected due to the complex nature of physical (in)activity with adults with a learning disability, as it is a comprehensive method of review that aims to map and summarise existing literature, which can be useful where best practices have not yet been defined.^16^ The purpose of this scoping review is to gain a broad understanding of the size and scope of literature investigating the role of support networks in promoting physical activity for adults with a learning disability.^17^ By identifying who the key actors are within these networks, the nature of their involvement and the relational and structural dynamics that shape participation, this review seeks to develop a broad understanding of using support networks to promote and facilitate physical activity to inform future research practices, as well as potentially policy making and related practices. In doing so, this review will contribute to the growing body of evidence calling for more inclusive and informed approaches to reducing health inequalities and improving well-being for adults with a learning disability.^8 18^ This scoping review will be guided by the Preferred Reporting Items for Systematic Reviews and Meta-Analyses Extension for Scoping Reviews Checklist (PRISMA-ScR).^19^ This review will be conducted in line with framework established by Peters *et al*.^20^ to reflect current best practice guidance for scoping reviews.^20^ We will also include a participatory element to this research, in which we will share preliminary findings with adults with a learning disability and members of their support networks to guide our synthesis of information to strengthen practical relevance and interpretation.

## Methods

### Eligibility criteria

The search terms were developed using the population, concept and context framework to reflect the scope of the review and inclusion criteria.

### Population

Adults (18 and older) with a learning disability, including those with multiple or profound disabilities.

The support networks of adults with a learning disability, including but not limited to family, caregivers, community organisations, peer support, advocacy groups, professional/formal support and informal support.

### Concept

The role, influence and integration of (formal and/or informal) support networks in relation to physical activity participation. Physical activity participation can be broadly conceptualised to include activities that require moving more. Randomised control trials or prescribed exercises (eg, clinical interventions or rehabilitative exercises) are not included in this review.

### Context

Physical activity, exercise, sport and active leisure across home, community, care and organised activity settings.

### Types of evidence sources

Qualitative, quantitative and mixed-methods research will also be considered for inclusion in this review. Due to the importance of cultural and policy context in support systems and opportunities for physical activity, only studies conducted in countries with established health and disability policies (eg, UK, USA, Australia, EU and Canada) will be included. Although the CRPD provides a global rights-based framework, its implementation varies substantially across countries.^21^ Therefore, studies conducted in countries where the sociocultural and policy contexts relating to adults with a learning disability differ significantly from those in the UK, particularly in terms of recognition of rights, inclusion practices and societal attitudes, will be excluded. This is to ensure culturally relevant findings; an important aspect to be considered when mapping literature.^19 22–24^ This review will only include literature published in English, as there are insufficient resources for papers to be translated from other languages. Furthermore, there is potential for terminology and context to be misinterpreted with translation, which may interfere with the findings of this review. Only studies published after 2010 will be included to ensure historical relevance.

### Search strategy

This search strategy aims to be inclusive of studies across disciplines. Therefore, a selection of health and social science databases will be used. To guide the conceptualisation of our review, an initial search was carried out in April 2025 to scope the expanse of the data available and refine search terms. An updated and complete search was undertaken in September 2026 to include literature from January 2010 to September 2026. Full search strategy can be found as an online supplemental material. All search results will be exported to EndNote and deduplicated, using the Endnote ‘remove duplicates’ function by ER, before being imported into Rayyan for screening. To maximise the likelihood of identifying relevant publications, a reference search will be conducted on all articles subjected to full-text screening, including a forward citation search. Grey literature will not be included.

### Source of evidence selection

The PRISMA-ScR checklist will be used and a flow diagram will be provided to increase the reproducibility and transparency of study selection.^19^ A selection checklist has been developed, based on the inclusion and exclusion criteria, to ensure consistency within the study selection process (included in online supplemental materials).

Identified sources will first be screened at the level of title by ER. All remaining articles will be screened at abstract level and finally a full-text review. A subset of data (20%) will be screened independently at abstract level and full-text level, by two reviewers, ER and JB using Rayyan software.^25^ Cohen’s kappa (κ) will be calculated to assess inter-rater reliability. If interrater reliability is achieved (0.6–1, indicating substantial agreement), ER will independently review the remaining articles.^26 27^ Any disagreements will be resolved through discussions between ER and JB; in the case consensus cannot be reached, a third author (AP) will be included in discussions. The searching, screening and selection process may be modified or expanded during the review process, in the event that potentially relevant terms, concepts or sources of evidence emerge. An adapted PRISMA 2020 flow diagram will be included to depict the search and selection process^28^

### Data extraction

Data will be extracted to gather general information about the study and in relation to the research questions. A data extraction form will be created using Microsoft Excel, outlined below:

- General study information (title, author, date of publication, geographic location).
- Research aims.
- Sample.
- Methods and design.
- How was physical activity measured/defined.
- Key findings and general conclusions.
- Study limitations.
- Key points relating to research question 1:
  Who and what are the key drivers or limiters within support networks in relation to physical activity participation for adults with a learning disability?

- Key points relating to research question 2:
  How do those within and across support networks (eg, families, professional caregivers and advocacy groups) interact, collaborate and influence each other in promoting physical activity for adults with a learning disability?

- Key points relating to research question 3:
  How do adults with a learning disability engage in decision-making within their support networks in relation to physical activity, and to what extent do they have power and autonomy in these relationships?

- Other salient points.

A subset of data (20%) will be extracted by ER and JB to ensure consistency in the extraction process; any disagreements will be resolved through discussion. Following this, ER will extract data from remaining sources. Data extraction will be an iterative process whereby the data extraction form may be revisited and updated, particularly as knowledge and concepts emerge; discussions will be had between ER, JB and AP in the case of any iterations to the extraction process. The final completed extraction table will be shared within the publication of the scoping review.

### Data synthesis and presentation

Characteristics of included studies will be reported in tabular format. We will provide a narrative synthesis describing the data in relation to the review questions. Visuals, such as concept maps and figures may be used to present data; final decisions on data presentation will be made when the data has been charted and described.

### Patient and public involvement

This review protocol was conceptualised following an inclusive research PhD study undertaken by ER (supervised by AP, PF, SC and ES) in which an advisory group, made up of members with a learning disability, and their support teams, identified a knowledge gap in terms of the role of support networks to facilitate physical activity. This group will be involved in the consultation stage of this review, as well as creating accessible formats to disseminate findings of this review beyond academic publication. Once the data extraction tool is complete, preliminary findings will be shared with key stakeholders (in this case, adults with a learning disability and members of their support networks), to engage in conversations which may offer additional context, meaning and perspective to the narrative summary. Due to the participatory nature of the overarching research project, this process will be included to sustain epistemological alignment, increase methodological rigour and aid practical application of the work.^22 23 29^ We also invite comments and dialogue following the publication of this protocol, through social media networks, and will consider any interactions and suggestions for the publication of the final review.

### Ethics and dissemination

This scoping review protocol does not require ethical approval as no primary data have been collected or analysed. The findings of this review will be disseminated to key stakeholders and integrated into future research. We hope the publication of this protocol will initiate interactions with the wider academic community as well as key stakeholders, where feedback and comments could shape future work, including incorporating feedback into the full scoping review. It is anticipated that the findings will identify both enablers and barriers within formal and informal support systems, offering insights into the relational, structural and contextual factors that shape opportunities for engagement in physical activity for adults with a learning disability. Mapping the evidence base in this way will help to clarify existing knowledge and highlight gaps to inform the development of future research. The completed review will be submitted for publication in a peer-reviewed academic journal and disseminated through conference presentations and stakeholder networks, with accessible outputs developed to support knowledge exchange among practitioners, advocacy organisations and people with lived experience. For questions regarding research integrity, please contact the research integrity office at reo-governance@essex.ac.uk.

## Supplementary material

10.1136/bmjopen-2026-117713 online supplemental file 1

10.1136/bmjopen-2026-117713 online supplemental file 2
