# Supplementary material for: How do support networks influence physical activity participation among adults with a learning disability? A scoping review protocol
Source: BMJ Open. 2026 Oct 23;16(10):e117713. doi: 10.1136/bmjopen-2026-117713 (PMC13711560; doi:10.1136/bmjopen-2026-117713)
Supplement: Supplementary file 1 [file bmjopen-2026-117713.reviewer_comments.pdf]

PEER REVIEW HISTORY

BMJ Open publishes all reviews undertaken for accepted manuscripts. Reviewers are asked to complete a checklist review form and are provided with free text boxes to elaborate on their assessment. These free text comments are reproduced below.

ARTICLE DETAILS

Title (Provisional)

How Do Support Networks Influence Physical Activity Participation among Adults with a Learning Disability? A Scoping Review Protocol

Authors

Rossiter, Ellie; Pettican, Anna; Freeman, Paul; Black, James; Coetzee, Simone; Speed, Ewen

---

VERSION 1 - REVIEW

**Reviewer**	**1**
**Name**	**Petro-Petro, Jose**
**Affiliation**	**Universidad de Antioquia**
**Date**	**05-Jun-2026**
**COI**	**none**

Greetings. The submitted scoping review protocol shows considerable potential and addresses a highly interesting topic for public health with substantial social relevance. However, it presents some methodological inconsistencies when evaluated against the rigor frameworks currently recommended for conducting this type of review.

Below, I outline several aspects that should be considered and that suggest the need for major revisions:

Comments	Suggestions
The manuscript is based on the framework of Tricco et al, 2018 and PRISMA-ScR.	It is suggested to update the protocol under the framework established by Peters et al. 2022, “Best practice guidance and reporting items for the development of scoping review protocols”.<br>• (https://doi.org/10.11124/JBIES-21-00242).<br>Following the elements established within this framework will ensure coherence with PRISMA-ScR. Additionally, this

	methodological framework aims to describe how to develop a robust and detailed scoping review protocol and includes elements that could improve this protocol beyond what is already established.
I consider that the use of the framework proposed by Arksey & O'Malley (2005) is classic, acceptable, and still valid from a methodological perspective for presenting scoping reviews. However, these reviews have been evolving for many years, and more current frameworks have been proposed that seek to improve the presentation and completeness of reporting for this type of evidence synthesis. It would be good to consider these updates in your methodological framework for presenting the scoping review, which do not disregard the elements of the classic framework by Arksey & O'Malley but rather complement them. These updates in methodological frameworks align with and are more consistent with the PRISMA-ScR checklist.	It is also suggested to update the Arksey & O'Malley scoping review presentation framework with what has been established by Peters et al., 2015; 2020.<br>• (https://doi.org/10.1097/XEB.0000000000000050).<br>• (https://doi.org/10.11124/jbies-20-00167)<br>(Peters MDJ, Godfrey C, McInerney P, Baldini Soares C, Khalil H, Parker D 2017, Chapter 11: Scoping Reviews. In: Aromataris E, Munn Z (Editors). Joanna Briggs Institute Reviewer's Manual. The Joanna Briggs Institute, 2017)
Regarding the research question, scoping reviews do not use PICO terms because this methodology is more characteristic of systematic reviews with or without meta-analysis, or SPIDER methodological framework associated with qualitative syntheses.<br><br>Scoping reviews typically use the PCC model (Population, Concept, Context), which makes the search less restrictive and allows for obtaining much more information on the topic. "A scoping review will have a broader 'scope' with correspondingly less restrictive inclusion criteria. Based upon the PCC (Population, Concept and Context)" (https://jbi-global-wiki.refined.site/space/MANUAL/355862573/10.1.2+Scoping+reviews+compared+to+other+types+of+review). It should be noted that: "For instance, because scoping reviews are typically more concerned with identifying broader, more descriptive elements of the literature rather than answering specific analytical questions of effectiveness, the inclusion criteria of 'concept' and 'context' may be used instead of 'intervention' and 'comparator.' This is because scoping reviews do not aim to compare	I suggest using the PCC methodology, which is more appropriate for scoping reviews. This methodology applies to the research questions of the scoping review as well as to defining elements of the search strategy.<br><br>https://jbi-global-wiki.refined.site/space/MANUAL/355862573/10.1.2+Scoping+reviews+compared+to+other+types+of+review

interventions in terms of pooled results effectiveness, but to descriptively map and explain the nature of a body of evidence" (Peters et al., 2022).	
There should be no confusion in the analysis of data or information. It is mentioned that: "Data will be extracted, collated and then summarised descriptively using thematic analysis". A methodologically pure JBI scoping review does not conduct a thematic analysis per se (as thematic analysis belongs to qualitative synthesis or meta-ethnography). The JBI specifies that data are extracted, mapped, and presented in tabular or graphical form, accompanied by a descriptive narrative summary that aligns with the review objectives and questions.	I suggest revising the wording to clarify that a descriptive mapping of the content will be conducted rather than an in-depth inductive/deductive qualitative thematic analysis, or alternatively, specifying whether a basic descriptive content analysis will be used.

Reviewer 2

Name Tomsone, Signe

Affiliation Riga Stradins University

Date 10-Aug-2026

COI none

Dear Authors,

Thank you for the opportunity to read the scoping review protocol "How Do Support Networks Influence the Participation of Individuals with Learning Disabilities in Physical Activity?"

I find the protocol well written and structured, the methodology for review is clearly describes and explained. However, I have few minor comments:

1) Strength and limitations section points that literature from various disciplines will be included. Later, in description of Stage 2, it is more specific described that literature from health and social sciences data bases will be selected.

2) protocol includes terms "individuals with learning disabilities", "people with learning disabilities" but in the description of Stage 2, again, it is more clearly expressed that review will focus on adults.

3) Description of Stage 4 clearly presents some general information units for data extraction but in relation to three research questions, perhaps, they could be formulated more specific.

4) The time for preliminary study is reported (April 2025) but for the main review time frame is not mentioned.

5) In the Patient and public involvement section stated "This review was conceptualised...", perhaps, "review protocol" should be mentioned.

VERSION 1 - AUTHOR RESPONSE

Peer reviewer feedback	Author response
The manuscript is based on the framework of Tricco et al, 2018 and PRISMA-ScR.<br>It is suggested to update the protocol under the framework established by Peters et al. 2022, "Best practice guidance and reporting items for the development of scoping review protocols". (https://doi.org/10.11124/JBIES-21-00242).<br>Following the elements established within this framework will ensure coherence with PRISMA-ScR. Additionally, this methodological framework aims to describe how to develop a robust and detailed scoping review protocol and includes elements that could improve this protocol beyond what is already established.	We have updated the protocol to follow the framework established by Peters et al. 2022. This has included restructuring parts of the manuscript to allow for what we hope is a detailed and robust scoping review protocol.
It is also suggested to update the Arksey & O'Malley scoping review presentation framework with what has been established by Peters et al., 2015; 2020.	In updating the protocol framework, we have revised the presentation to reflect that established by Peters et al., 2015; 2020
I suggest using the PCC methodology, which is more appropriate for scoping reviews. This methodology applies to the research questions of the scoping review as well as to defining elements of the search strategy.	We have combined and refined the elements from the previous combination of the SPIDER and PICO frameworks to a more succinct PCC framework. We feel this more effectively captures the concept of this scoping review.
I suggest revising the wording to clarify that a descriptive mapping of the content will be conducted rather than an in-depth inductive/deductive qualitative thematic analysis, or alternatively, specifying whether a basic descriptive content analysis will be used.	We have reconsidered how we will synthesise and report on data. We have revisited wording to more accurately describe the process of extracting the data, mapping and presenting in tabular/graphical form, accompanied by a descriptive narrative summary that aligns with the review questions and objectives.

1) Strength and limitations section points that literature from various disciplines will be included. Later, in description of Stage 2, it is more specific described that literature from health and social sciences data bases will be selected.	This has prompted reflection on the databases used- we have reconsidered our wording to more accurately describe the health and social sciences data bases used.
2) Protocol includes terms "individuals with learning disabilities", "people with learning disabilities" but in the description of Stage 2, again, it is more clearly expressed that review will focus on adults.	Thank you for this interesting point. We have considered wording throughout and in many places replaced 'people' or 'individuals' to explicitly reflect our focus on adults with a learning disability. This has also prompted further consideration on our language and we have consistently used the term 'adults with a learning disability' or 'people with a learning disability' in line with
3) Description of Stage 4 clearly presents some general information units for data extraction but in relation to three research questions, perhaps, they could be formulated more specific.	We hope that the extraction tool will allow for overall characteristics to be reported as well as more specific information relating directly to the research questions, which will allow us to potentially map data relating to RQs with general characteristics.
4) The time for preliminary study is reported (April 2025) but for the main review time frame is not mentioned.	Time frame added in search strategy (in text and supplementary file)
5) In the Patient and public involvement section stated "This review was conceptualised...", perhaps, "review protocol" should be mentioned.	Added to now read: *"This review protocol was conceptualised..."*
