# Supplementary material for: How do support networks influence physical activity participation among adults with a learning disability? A scoping review protocol
Source: BMJ Open. 2026 Oct 23;16(10):e117713. doi: 10.1136/bmjopen-2026-117713 (PMC13711560; doi:10.1136/bmjopen-2026-117713)
Supplement: Supplementary file 2 [file bmjopen-16-10-s001.docx]

**Inclusion Checklist:**

| **How Do Support Networks Influence Physical Activity Participation among Adults with a Learning Disability? A Scoping Review Protocol** | | | | |
| --- | --- | --- | --- | --- |
| For each study, check whether it meets the inclusion/exclusion criteria below. Only include studies that meet all inclusion criteria and do not meet any exclusion criteria**.** | | | | |
|  | Criteria | yes | no | Notes |
| **General eligibility** | Is the study published within the selected time frame (e.g., January 2010–September 2026)? |  |  |  |
|  | Is the study available in English? |  |  |  |
|  | Is the study peer-reviewed? |  |  |  |
| **Population** | Does the study explicitly focus on **adults** with a learning disability**?** |  |  |  |
|  | If the study includes multiple disabilities, does it provide subgroup data for people with a learning disability? |  |  |  |
| **Support networks**  ***(****Exclude if****:*** *The study focuses* *only on personal motivation or individual factors(e.g., self-efficacy) without discussing support networks.)* | Does the study examine the role of support networks (family, caregivers, advocacy groups, community organisations, peer support)? |  |  |  |
|  | Does it explore interactions, collaboration, or overlaps between different support networks? |  |  |  |
|  | Does it investigate the role of support network interventions(e.g., caregiver training, community-based programs, professional support)? |  |  |  |
| **Physical activity**  *(Exclude if****:*** *The study focuses only on clinical rehabilitation (e.g., physiotherapy) without discussing physical activity in a broader sense).* | Does the study explicitly focus on physical activity participation? |  |  |  |
|  | Does it include structured(sports programs, exercise classes) or unstructured(walking, play, leisure activities) physical activity? |  |  |  |
| **Research type**  *(Exclude if: The study is an* *opinion piece, editorial, or commentary* *with no empirical data. Exclude all literature reviews)****.*** | Is the study qualitative, quantitative, or mixed-methods research? |  |  |  |
|  | If a review, does it contribute new insights, synthesise evidence, or highlight research gaps? |  |  |  |
| **Context**    *(Exclude if****:*** *The study only focuses on policy frameworkswithout real-world application to support networks and physical activity).* | Does the study come from a geographical region relevant to the UK (e.g., high-income countries with relevant health and disability policies)? |  |  |  |
|  | If the study discusses policy or practice, does it provide applicable insights into support networks and physical activity? |  |  |  |
| **Final decision** | Does this study meet all inclusion criteria and should therefore be included in the review? |  |  |  |
| **Notes for further discussion** |  | | | |
