# Supplementary material for: How do support networks influence physical activity participation among adults with a learning disability? A scoping review protocol
Source: BMJ Open. 2026 Oct 23;16(10):e117713. doi: 10.1136/bmjopen-2026-117713 (PMC13711560; doi:10.1136/bmjopen-2026-117713)
Supplement: Supplementary file 3 [file bmjopen-16-10-s002.docx]

Search strategy

| Database | Search date | Search string | Records retrieved |
| --- | --- | --- | --- |
| Scopus (Elsevier) | 08/09/2026 | ( TITLE-ABS-KEY ( "Learning disability" ) OR TITLE-ABS-KEY ( "learning difficulty" OR "intellectual disability" OR "down syndrome" ) AND TITLE-ABS-KEY ( Support* ) OR TITLE-ABS-KEY ( care* OR help* OR famil* OR parent* OR "decision making" ) AND TITLE-ABS-KEY ( "Physical activity" ) OR TITLE-ABS-KEY ( sport* OR exercis* OR fitness ) ) AND PUBYEAR > 2009 AND ( LIMIT-TO ( LANGUAGE , "English" ) ) | 1942 |
| Web of Science Core Collection (Clarivate) | 08/09/2026 | (TOPIC) “Learning disability” or "learning difficulty" or "intellectual disability" or "down syndrome" AND (TOPIC)  Support* or care* or help* or famil* or parent* or "decision making" AND (TOPIC) “Physical activity” or sport* or exercis* or fitness   Limiters: English language only, published between 1^st^ January 2010 and 8^th^ September 2026 | 1023 |
| Pubmed (NLM) | 08/09/2026 | (("Learning disability"[Title/Abstract] OR "learning difficulty"[Title/Abstract] OR "intellectual disability"[Title/Abstract] OR "down syndrome"[Title/Abstract]) AND ("support*"[Title/Abstract] OR "care*"[Title/Abstract] OR "help*"[Title/Abstract] OR "famil*"[Title/Abstract] OR "parent*"[Title/Abstract] OR "decision making"[Title/Abstract]) AND ("Physical activity"[Title/Abstract] OR "sport*"[Title/Abstract] OR "exercis*"[Title/Abstract] OR "fitness"[Title/Abstract])) AND (2010/1/1:2026/9/8[pdat]) | 614 |
| Wiley Online Library (Wiley) | 08/09/2026 | “Learning disability” OR "learning difficulty" OR "intellectual disability" OR "down syndrome"" in Keywords and "Support* OR care* OR help* OR famil* OR parent* OR "decision making"" anywhere and ""Physical activity" OR sport* OR exercis* OR fitness" in Keywords  Limiters: published between 1^st^ January 2010 and 8^th^ September 2026 | 118 |
| APA PsycArticles;CINAHL Ultimate;APA PsycInfo;SPORTDiscus with Full Text (EBSCOhost) | 08/09/2026 | SU (“Learning disability” OR "learning difficulty" OR "intellectual disability" OR "down syndrome") AND SU (Support* OR care* OR help* OR famil* OR parent* OR "decision making") AND SU ("Physical activity" OR sport* OR exercis* OR fitness)  Limiters: English language, 01/01/2010 - 09/08/2026 | 378 |
| **Total before duplicates removed** | | | **4075** |

This review will only include literature published in English, due to the potential for terminology and context to be misinterpreted with translation, which may interfere with the findings of this review. Only studies published after 2010 will be included to ensure historical relevance. Therefore, limiters and/or filters have been applied within database searches.

Once all articles have been screened and title and abstract stage, a reference list search will be conducted by ER on all articles subjected to full-text screening, including a forward citation search.

Articles will be exported into endnote and deduplicated by ER using endnote ‘remove duplicates’ function.
